# Optimization and Characterization of Novel ALCAM-Targeting Antibody Fragments for Transepithelial Delivery

**DOI:** 10.3390/pharmaceutics15071841

**Published:** 2023-06-27

**Authors:** Aline Bauer, Sven Klassa, Anja Herbst, Cristina Maccioni, William Abhamon, Noria Segueni, Yulia Kaluzhny, Morgan Campbell Hunter, Cornelia Halin

**Affiliations:** 1 Institute of Pharmaceutical Sciences, ETH Zurich, 1-5/10 Vladimir-Prelog-Weg, 8093 Zurich, Switzerland; 2 Artimmune SAS, 13 Avenue Buffon, 45100 Orleans, France; 3 MatTek Corporation, 200 Homer Avenue, Ashland, MA 01721, USA

**Keywords:** monoclonal antibody fragment, ALCAM, topical treatment, asthma, cornea

## Abstract

Activated leukocyte cell adhesion molecule (ALCAM) is a cell adhesion molecule that supports T cell activation, leukocyte migration, and (lymph)angiogenesis and has been shown to contribute to the pathology of various immune-mediated disorders, including asthma and corneal graft rejection. In contrast to monoclonal antibodies (mAbs) targeting ALCAM’s T cell expressed binding partner CD6, no ALCAM-targeting mAbs have thus far entered clinical development. This is likely linked with the broad expression of ALCAM on many different cell types, which increases the risk of eliciting unwanted treatment-induced side effects upon systemic mAb application. Targeting ALCAM in surface-exposed tissues, such as the lungs or the cornea, by a topical application could circumvent this issue. Here, we report the development of various stability- and affinity-improved anti-ALCAM mAb fragments with cross-species reactivity towards mouse, rat, monkey, and human ALCAM. Fragments generated in either mono- or bivalent formats potently blocked ALCAM–CD6 interactions in a competition ELISA, but only bivalent fragments efficiently inhibited ALCAM–ALCAM interactions in a leukocyte transmigration assay. The different fragments displayed a clear size-dependence in their ability to penetrate the human corneal epithelium. Furthermore, intranasal delivery of anti-ALCAM fragments reduced leukocyte infiltration in a mouse model of asthma, confirming ALCAM as a target for topical application in the lungs.

## 1. Introduction

Activated leukocyte cell adhesion molecule (ALCAM) is a cell adhesion molecule of the immunoglobulin superfamily and is expressed by various cell types, including neurons, keratinocytes, leukocytes, and endothelial cells [1,2]. ALCAM engages in low-affinity homophilic interactions that stabilize cell–cell contacts [1] as well as higher-affinity interactions with CD6 expressed on T cells [3]. In endothelial cells, ALCAM–ALCAM interactions were shown to contribute to (lymph)angiogenic processes in vitro and pathologic angiogenesis and developmental lymphangiogenesis in vivo [4,5,6,7]. ALCAM–ALCAM interactions were also shown to support the in vitro transmigration of monocytes [8,9] across the blood vascular endothelium as well as the transmigration of dendritic cells (DCs) across the lymphatic endothelium [7]. Consequently, the migration of DCs from the lungs to lung-draining lymph nodes was reduced in ALCAM-deficient mice [4]. Moreover, ALCAM expressed by DCs is well known to support T cell activation by forming a costimulatory interaction with T-cell-expressed CD6 [3,7,10]. Similarly to CD6-deficient mice [11,12,13], ALCAM-deficient mice were found to develop a reduced T cell response in vivo, as recently demonstrated in mouse models of asthma, atopic dermatitis, and food allergies [10,14,15]. Given the contribution of ALCAM to (lymph)angiogenesis, leukocyte trafficking, and T cell activation, therapeutic blockade of ALCAM with monoclonal antibodies (mAbs) could represent a promising approach for treating immune-mediated inflammatory disorders that typically involve all of these processes. For instance, intranasal delivery of rabbit polyclonal anti-ALCAM antibodies significantly reduced the inflammatory response in a murine asthma model [10]. Our group recently showed, in a mouse model of high-risk corneal transplantation, that allograft rejection was significantly reduced upon systemic treatment with a monoclonal anti-ALCAM antibody [7]. However, in contrast to CD6-targeting antibodies [16,17], no antibodies targeting ALCAM have thus far entered clinical development for the treatment of immune-mediated inflammatory diseases.

Currently, most approved therapeutic antibodies are delivered either intravenously or by subcutaneous injection. Considering the broad expression of ALCAM on many cell types, a systemic treatment targeting ALCAM could lead to serious side effects. Therefore, local delivery via the topical route to the tissue of interest, such as the cornea or lungs, is desirable for achieving high local concentrations while sparing other body sites. However, the corneal epithelium and the large, mucus-covered surface of the lungs represent considerable barriers for the topical administration of protein-based therapeutics, particularly large (150 kDa) full-length mAbs [18,19,20]. In fact, several studies have shown that, in comparison to conventional full-length mAbs, antibody fragments display better penetration across the corneal epithelium [21,22,23,24] or the lung epithelium [25,26,27]. In addition to the antibody size, stability is another important determinant of transepithelial delivery. Because of the low transcorneal penetration efficiency, therapeutic proteins delivered as eye drops require high stability and solubility to achieve the required dose in the tissue [21,28]. Similarly, inhaled therapeutic antibodies for topical drug delivery to the lungs require high stability, since the antibodies are exposed to significant physical stress during aerosolization [29]. Currently, several inhaled antibody fragments targeting, e.g., thymic stromal lymphopoietin (TSLP), tumor necrosis factor receptor 1 (TNFR-1), and interleukin-13 (IL-13), are under clinical investigation for the treatment of pulmonary diseases, particularly asthma [19].

In this study, we set out to generate anti-ALCAM antibody fragments with high affinity, stability, and solubility for convenient storage and maximal dosing via the topical route. As a starting antibody, we chose the ALCAM-blocking antibody IF8 [30], which we previously demonstrated to be efficacious in preventing corneal allograft rejection in mice upon systemic administration [7]. We here describe the generation and in vitro bioanalytical and functional characterization of the affinity-matured and stability-optimized antibody clone V2D7 produced in different antibody fragment formats. Finally, we tested two of the newly developed antibody fragments as well as the parent antibody IF8-Fc in vivo in a mouse model of allergic asthma to further validate ALCAM as a disease target upon topical application.

## 2. Materials and Methods

### 2.1. Recombinant ALCAM Protein

Murine ALCAM (50005-M08H-100, Sino Biological, Beijing, China), monkey ALCAM (10027-AL-100, R&D systems, Minneapolis, MN, USA), and rat ALCAM (80221-R08H-50, Sino Biological, China) were purchased commercially. Human ALCAM (28–501)-6xhistidine tag (hALCAM) expressing all five extracellular immunoglobulin domains of ALCAM was produced in-house in Chinese hamster ovary (CHO) cells and purified by affinity chromatography using cOmplete Ni-NTA agarose resin (5893682001, Roche, Basel, Switzerland), according to Strassberger et al. [31]. The outermost extracellular domain of hALCAM V1 was produced in-house using a similar approach to that mentioned above (hALCAM V1 (28–137)-Avi tag-6x-histidine tag). The gene strand featured a C-terminal BirA target sequence (Avi tag, GLNDIFEAQKIEWHE), allowing for site-specific biotinylation, and was carried out by using the BirA enzyme in BirA buffer (10 mM Tris pH 7.5, 200 mM NaCl, 5 mM MgCl_2_), following the protocol described by Fairhead et al. [32].

### 2.2. Cloning, Expression, Purification, and Characterization of Proteins

Stable CHO cell lines producing KSF-Fc (specific to hen-egg lysozyme; used as isotype control) and IF8-Fc (specific to ALCAM) were generated in-house as described by Willrodt et al. [7]. All other antibodies or recombinant proteins were produced in CHO cells using transient gene expression, according to previously described procedures [33]. All gene sequences were cloned into HindIII/NotI of pcDNA3.1(+) plasmids (GenScript, New Jersey, NJ, USA). Briefly, CHO cells were cultured in powerCHO-2CD (BE12-771Q, Lonza, Basel, Switzerland), and proteins were produced in proCHO-4 (BE12-029Q, Lonza). All media were supplemented with 1X antibiotic-antimycotic (15240-096, Gibco, Thermo Fisher Scientific, Waltham, MA, USA), 1X proHT supplement (BE17-855E, Lonza), and 8 mM ultraglutamine (BE17-605E/U1, Lonza). The transfection agent polyethylenimine (25K PEI, 23966-1, Polysciences, Warrington, PA, USA) was used at 1% with a PEI to plasmid ratio of 3:1. The product was purified from the cell culture medium by affinity chromatography using a Protein A affinity column. A low pH, 0.1 M glycine buffer (pH 2.5–3.0) was used for protein elution and neutralized with 1 M Tris HCl buffer (pH 7.4). Purified proteins were dialyzed overnight into PBS, passed through 0.22 µm filters, and stored at −80 °C after snap-freezing in liquid nitrogen. Purified proteins were analyzed by SDS-PAGE and size-exclusion chromatography (SEC, Superdex 75 or 200 depending on the size of the protein, GE Healthcare, Chicago, IL, USA) on an ÄKTA pure (GE Healthcare).

### 2.3. Antibody Format Design

Antibodies were produced in CHO cells in various formats, including single-chain variable fragment (scFv), diabody (db), single-chain diabody (sc-db), tandem-scFv (ta-scFv), and mini-antibody (mini-ab), as specified in Table 1. The mini-ab is composed of two scFvs, each fused via a linker to a dimerization domain (leucine zippers derived from the yeast transcription factor GCN4 [34,35]).

### 2.4. Sequence Optimization Generating OPT

To generate the framework-optimized scFv OPT, the complementarity-determining regions (CDRs) and important framework residues of IF8 (donor) were grafted onto a stabilized acceptor framework (FW), namely an ESBA FW, described in the following patent [36] (Figure S1A). Important donor and acceptor residues were identified as in Ewert et al. [37]. In the case of the variable light chain (V_L_), the resulting graft was further mutated to that of the nearest germline sequence (IGLV3-21*02), as defined in the IMGT Repertoire [38]. The SAbPred Therapeutic Antibody Profiler (TAP) was used for comparing IF8 and OPT amino acid sequences against developability guidelines derived from >500 clinical-stage therapeutics [39] (Figure S1B).

### 2.5. Construction of the Affinity Maturation Library

The OPT scFv clone was used as the template for the construction of the affinity maturation library following the protocol described by Villa et al. [40]. Sequence variability was first introduced in the V_H_ or V_L_ of CDR1 by PCR using partially degenerate primers synthesized by Microsynth (Balgach, Switzerland) (Table 2). Random mutations were generated at positions 31, 32, and 33 of the V_H_ and 31, 31a, and 32 of the V_L_ of CDR1 (antibody residues numbered according to Cox et al. and Tomlinson et al. [41,42]). First, three fragments were obtained by PCR from the OPT clone plasmid using the primer pairs a/b, c/d, and e/f. After gel purification, the segments were assembled by PCR and further amplified using primers a/f. The assembled fragments were double-digested with NcoI/NotI and cloned into the NcoI/NotI-digested expression vector pHEN1. The resulting ligation mixture was purified and electroporated into fresh electrocompetent TG1 bacteria (prepared according to Villa et al. [40]). Electroporated bacteria were streaked out on 2xYT-agar plates and incubated at 30 °C overnight. On the next day, bacteria were rescued with 2xYT-10% glycerol, used for phage production according to the standard protocol with the helper phage VCS-M13 (Stratagene, San Diego, CA, USA) [43], and stored as glycerol stocks. To examine whether the library was successfully built with the intended residues randomized in the CDR1 and CDR2 loops, 10 random clones were picked and analyzed by colony sequencing (performed by Microsynth). In a second step, sequence variability was introduced into the V_H_ or V_L_ of CDR2 based on the CDR1 affinity-matured scFv clone V2D7 by PCR using partially degenerate primers (Table 2, synthesized by Microsynth), using the same protocol as mentioned above. Random mutations were generated in CDR2 at positions 50, 52a, 53, and 56 of the V_H_ and 50, 52, and 53 of the V_L_.

### 2.6. Selection of Affinity-Matured Anti-ALCAM Antibodies by Phage Display

Fully human affinity-matured scFv clones specific to the extracellular domain of hALCAM were isolated from the affinity-maturation phage library, following the protocol described by Viti et al. [43] and the affinity-maturation scheme explained in Table 3. Briefly, biotinylated hALCAM V1 was immobilized on 8 wells of a streptavidin-coated plate (11645692001, Roche) and incubated overnight at 4 °C. Wells were blocked with 2% milk–PBS for 2 h at room temperature (RT). Subsequently, 125 µL/well of scFv-displaying phages in 2% milk–PBS were added and incubated for 30 min at RT while shaking at 140 rpm, followed by 1.5 h incubation at RT without agitation. In competitive selections, phage particles were added to the antigen-coated wells in the presence of competing antibodies (see Table 3). Unbound phages were washed with 0.1% Tween in PBS (20–30 times) and subsequently in PBS (20–30 times). Bound phages were eluted with 125 µL of 100 mM triethylamine (471283, Sigma-Aldrich, St. Louis, MO, USA) in each well and pooled elute was transferred to 0.5 mL of 1 M Tris-HCl pH 7.4 for neutralization. Eluted phages were then used for infection of exponentially growing *E. coli* TG1 (kindly provided by the group of Prof. Dr. Dario Neri, ETH, Zurich, Switzerland) and incubated for 40 min at 37 °C. After preparing serial dilutions on agar plates (2xYT, 0.1% glucose, 100 µg/mL ampicillin), the remaining bacteria were spun down for 10 min at 3300 g, spread on agar plates and incubated at 30 °C overnight. On the next day, bacteria were rescued with 2xTY-10% glycerol and stored as glycerol stocks.

### 2.7. Bacterial Expression of Phagemid scFv

Individual bacterial colonies were inoculated in 2xYT media supplemented with 0.1% glucose and 100 µg/mL ampicillin and grown for 3 h at 37 °C. Expression of scFv was induced by adding 1 mM IPTG (LU5002-005, LubioScience, Zürich, Switzerland) and incubating overnight at 37 °C while shaking. Plates were spun down at 1800× *g* for 10 min, and supernatants containing soluble antibody fragments were collected and screened by ELISA and surface plasmon resonance (SPR) for binding to recombinant ALCAM protein.

### 2.8. ELISA Screening of Bacterial Supernatant Expressing scFv

A 96-well plate (Nunc, Rochester, NY, USA) was coated overnight at 4 °C with hALCAM (5 μg/mL) in PBS. The plate was blocked with 4% milk–PBS for 2 h at RT. Subsequently, 20 µL of anti-myc tag murine antibody (clone 9E10, 0.8 µg/mL [44]) diluted in PBS containing 5% milk powder and 80 µL of bacterial supernatant was added to each well. After 2 h of incubation at RT, 100 µL of antimouse IgG horse radish peroxidase (HRP) (1:2000, A2554, Sigma-Aldrich) diluted in 2% milk–PBS was added to each well. Peroxidase activity was detected using 100 µL TMB substrate (77248, Biolegend, San Diego, CA, USA), and the colorimetric reaction was stopped by adding 50 µL of 1 M H_2_SO_4_, followed by absorbance measurements at 450 nm and 570 nm using a plate reader (Tecan, Männedorf, Switzerland).

### 2.9. SPR-Based Screening of scFv-Expressing Bacterial Supernatants

For the screening of affinity-matured clones, bacterial supernatants containing scFv clones that were positive in the ELISA screening were analyzed by performing SPR (BiaCore3000, GE Healthcare) to detect clones with an improved dissociation constant k_off_. The bacterial supernatants were first filtered in a 96-well plate (MSGVN2210, Millipore, Burlington, MA, USA) and a plate covered with a septa cover (29192561, Cytiva, Marlborough, MA, USA). Binding to hALCAM coated on a CM5-sensor chip at a density of 800 RU was analyzed using a flow rate of 10 µL/min.

### 2.10. CD6 Competition ELISA

A CD6 competition ELISA was performed according to Willrodt et al. [7]. Briefly, a 96-well plate (Nunc) was coated overnight at 4 °C with hALCAM (5 µg/mL) in PBS. The plate was blocked with 2% milk–PBS and subsequently incubated for 2 h at RT with a fixed amount of murine CD6-Fc (0.25 μg/mL, 727-CD, R&D Systems) and decreasing concentrations of antibody in PBS (2000 nM to 0.002 nM). Bound CD6-Fc was detected using HRP-coupled antihuman IgG antibody (A2554, Sigma-Aldrich). Peroxidase activity was detected by adding 100 µL of TMB substrate (77248, Biolegend), and the colorimetric reaction was stopped by adding 50 µL of 1 M H_2_SO_4_, followed by absorbance measurements at 450 nm and 570 nm using a plate reader (Tecan).

### 2.11. ELISA to Determine Species Cross-Reactivity

A 96-well plate (Nunc) was coated overnight at 4 °C with human, mouse, rat, or monkey ALCAM (5 μg/mL) in PBS. The plate was blocked with 2% milk–PBS for 2 h at RT. Subsequently, the plate was incubated with decreasing concentrations of scFv V2D7 in PBS (1:3 dilutions from 1000 nM to 0.051 nM) for 2 h at RT. Bound antibody was detected using HRP-coupled protein A (689202, Biolegend). Peroxidase activity was detected using TMB substrate (77248, Biolegend), and the colorimetric reaction was stopped using 1 M H_2_SO_4_, followed by absorbance measurements at 450 nm and 570 nm using a plate reader, as described above.

### 2.12. Affinity Measurements of Purified Antibodies by SPR

The affinity of antibodies was analyzed by SPR on a BiaCore3000 (GE Healthcare) using a CM5-sensor chip (GE Healthcare) coated with hALCAM at a density of 800 RU and a flow rate of 10 µL/min. Binding curves were analyzed with BIAevaluation 3.2 software (GE Healthcare). For cross-species reactivity, the chips were coated with human, murine, or monkey ALCAM following the manufacturer’s protocol.

### 2.13. Chemical and Thermal Stability

Eleven dilutions (0 M–8 M) of guanidine hydrochloride (GdnHCl, G3272, Sigma-Aldrich) in PBS were prepared. Subsequently, 15 µL of each GdnHCl dilution was mixed with 15 µL of antibody, diluted at 0.2 mg/mL in PBS and left overnight at RT to equilibrate. Capillaries (Tycho NT.6, TY-C001, Nanotemper, Munich, Germany) were loaded with the sample, and stability was measured by nano differential scanning fluorimetry (nanoDSF, NanoTemper) (350/330 nm absorbance ratios) with a thermal ramp rate of 30 °C per minute. The chemical stability, or chemical denaturation point (C_m_), was defined as the turning point in the plot representing a 350/330 nm ratio against the GdnHCl concentration. The thermal stability (T_m_) was determined using the 0 M GdnHCl sample (i.e., 0.2 mg/mL antibodies in PBS).

### 2.14. Stability Studies

Antibodies concentrated to 60 mg/mL in PBS (pH 7.4) were incubated for 1 day, 3 months, or 6 months at various temperatures (4 °C, RT, 37 °C, −80 °C after snap-freezing in liquid nitrogen). Samples were thawed at RT and visually observed for the presence of aggregates, spun down for 10 min at 16,000× *g*, and absorbance at 280 nM was measured. The purity and biological functionality of the antibodies were assessed by SEC and the CD6 competition ELISA.

### 2.15. Solubility

To investigate the solubility, antibodies were concentrated with a Vivaspin^®^ 15R Centrifugal Concentrator with MWCO 10,000 (VS15RH01, Sartorius, Göttigen, Germany) up to the concentration at which visible protein aggregation occurred. The concentrated antibodies were left at RT overnight. Then, the following day, they were centrifuged at 16,000× *g* for 10 min, and the concentration in the soluble fraction was determined.

### 2.16. Culture of Immortalized Murine Lymphatic Endothelial Cells

Conditionally immortalized murine lymphatic endothelial cells (imLECs) expressing a heat-labile version of the large T antigen [45] were cultured in media containing 40% DMEM (low glucose), 40% F12-Ham, 20% FBS, 1X antibiotic-antimycotic solution (all from Gibco), 56 µg/mL heparin (H3149, Sigma-Aldrich), 10 µg/mL endothelial cell growth supplement (211-GS, AbD Serotec, Kidlington, UK), and 2 nM L-glutamine (25030-024, Invitrogen, Waltham, MA, USA). Dishes were coated with 10 µg/mL collagen type I (5005-B, Advanced Biomatrix, Carlsbad, CA, USA) and 10 µg/mL fibronectin (Millipore). For expansion, imLECs were cultured at 33 °C in media supplemented with 1 U/mL murine interferon-gamma (IFNγ, 313-05, Peprotech, Rocky Hill, NJ, USA) to induce large T-antigen expression [45]. Around 48–72 h prior to the transmigration assay, imLECs were seeded and grown to confluence at 37 °C in media without IFNγ.

### 2.17. Generation of Bone Marrow-Derived DCs

Bone marrow (BM) was isolated from the tibias and femurs of CD11c-YFP mice [46], as previously described [47]. Red blood cells were lysed with ACK buffer (BD Biosciences, 555899). BM-derived DCs were cultured in DC medium containing RPMI 1640 (Sigma-Aldrich), 10% FBS (Invitrogen), 1% antibiotic-antimycotic, 15 mM HEPES, 1 mM sodium pyruvate, 2 mM L-glutamine (all from Gibco), 50 µM β-mercaptoethanol (Sigma-Aldrich), and 80 ng/mL GM-CSF (derived from the supernatant of myeloma cells (X63 Ag8.653) transfected with murine GM-CSF cDNA [48]). Between days 7 and 9, the floating cell fraction containing immature DCs was transferred into tissue-culture-treated dishes (TPP) in DC medium supplemented with 0.1 µg/mL LPS (ALX-581-009-L002, Enzo Life Sciences, Farmingdale, NW, USA) to induce DC maturation. After 24 h, the floating cells were harvested and used for the experiments. Animals were housed and experiments were performed under specific pathogen-free conditions. All experiments were approved by the Cantonal Veterinary Office Zurich under Project License ZH239/19.

### 2.18. In Vitro DC Transmigration Assay

Approximately 10^5^ imLECs were seeded onto the upper side of transwell membrane inserts with a 5 µm pore size (TCS004024, Jet Biofil, Guangzhou, China) coated with 10 µg/mL collagen type I (5005-B, Advanced Biomatrix) and 10 µg/mL fibronectin (FC010, Millipore) and grown to confluence for 2 days at 37 °C. On the day of the assay, the transwell inserts were placed into a new 24-well plate. imLEC monolayers and CD11c-YFP DCs were separately preincubated for 30 min at 37 °C with ALCAM-blocking antibody fragments in imLEC media (final concentrations 0.01, 0.1, 1, 10 nM). Following this blocking step, 5 × 10^4^–10^5^ BM-DCs in 100 µL of imLEC media were added to the upper well of the transwell insert, and 600 µL of imLEC media containing CCL21 (100 ng/mL, 250-13, PeproTech) was added to the bottom well underneath the transwell insert. Transwell plates were incubated at 37 °C for 4–5 h, and subsequently, the media in the bottom well was collected and the percentage of YFP-positive DCs that had transmigrated compared to the input was determined by flow cytometry. In the DC transmigration assay performed with media from the corneal penetration assay, medium was collected from the lower chamber of the corneal epithelial cell penetration assay and diluted 1:4 in imLEC media. A total of 100 µL of the resulting mixture was added to the upper chamber of imLEC-coated transwells and incubated for 30 min at 37 °C to allow for ALCAM blocking. Subsequently, 5 × 10^4^–10^5^ YFP+ BM-DCs were added to each transwell, and the experiment was performed as described above.

### 2.19. Flow Cytometry

All flow cytometry samples were recorded on a CytoFlex S instrument (Beckman Coulter, Brea, CA, USA), and data were analyzed using FlowJo software version 10.8.1 (BD Life Sciences, Franklin Lakes, NJ, USA).

### 2.20. Flow Cytometry Detection of ALCAM Expression by Human Corneal Epithelial Cells

Membranes of two inserts covered with human corneal epithelial cells (COR-100, Mattek, Ashland, MA, USA) were cut into pieces with microscissors, incubated with collagenase IV (4 mg/mL in PBS, 17104019, Life Technologies, Carlsbad, CA, USA) for 40 min at 37 °C, and filtered through a 40 µm cell strainer. Samples were preincubated for 10 min with anti-CD16/CD32 (101302, Biolegend) to reduce nonspecific staining. Dead cells were excluded from the analysis by staining with the Zombie Aqua Fixable Viability kit (1:500, 423102, Biolegend). Samples were stained with anti-ALCAM IF8-Fc-AF647 (2 µg/mL, in-house) or with the isotype control KSF-Fc-AF647 (2 µg/mL, in-house) for 20 min before analysis by flow cytometry.

### 2.21. Corneal Penetration Model

The 3D corneal tissue models (Mattek Corporation, EpiCorneal™ COR-100) were cultured according to the manufacturer’s instructions. On the day of the assay, tissues were transferred to a new 24-well plate containing 300 µL media (COR-100-MM, Mattek) and left to equilibrate for 30 min at 37 °C. Tissues were moved to the next well, and 50 µL antibody solution (IF8-Fc, V2D7 scFv and V2D7 db at 500 nM) or PBS was added to the top of each tissue. After 6 h of incubation at 37 °C, the medium in the bottom well was collected. Immediately afterwards, the transepithelial electrical resistance (TEER) was measured using an epithelial volt-ohm meter EVOM 3 (World Precision Instruments, Sarasota, FL, USA) according to the manufacturer’s instructions. Subsequently, the tissue barrier integrity was analyzed using the Lucifer yellow (LY) leakage assay according to the manufacturer’s instructions. The signal was measured on a spectrophotometer (450/528 nm, Infinite 200, Tecan). Only data points from corneal models with an intact tissue barrier were included in the analysis.

### 2.22. AlphaLISA

AlphaLISA was performed according to the manufacturer’s instructions (PerkinElmer, Waltham, MA, USA). In brief, 10 µL of 10 nM hALCAM V1 diluted in 1X AlphaLISA buffer (AL000C, PerkinElmer) was added to wells of a 96-well plate (6002350, PerkinElmer). For the standard curves, dilutions of antibodies (IF8-Fc, diabody V2D7, scFv V2D7) were prepared in COR-100 media (final concentrations 15 nM to 0.23 nM, 1:2 dilutions). For the following preparation, 10 µL from standard curve samples or from supernatants from the corneal penetration assay or blank COR-100 media was added in duplicate to each well. The plate was incubated for 60 min at RT. Solutions of anti-6Xhis acceptor beads (AL178C, PerkinElmer) and protein A donor beads (AS102D, PerkinElmer) were prepared at 80 µg/mL (4X), and 10 µL of each was added per well. The plate was incubated for 60 min at RT. Finally, the signal was acquired on a spectrophotometer (SpectraMax Paradigm, Molecular Devices, San Jose, CA, USA) using the AlphaLISA cartridge (excitation 680 nm, emission 570 nm, excitation time 40 ms, integration time 80 ms).

### 2.23. Allergic Asthma Study

The allergic asthma study was performed by Artimmune (Orleans, France) according to their internal protocol. Prior to the start of treatment, animals were randomized into equal groups based on body weight. Briefly, BALB/c female mice (9 weeks) were immunized by intraperitoneal injection of 20 µg ovalbumin (OVA) (chicken egg albumin fraction V, Sigma-Aldrich) and 2 mg of aluminum hydroxide gel (12261, Serva, Heidelberg, Germany) on days 0, 7, and 14. Mice were challenged daily between days 21 and 24 with 10 µg OVA in PBS applied by the intranasal route. Antibody treatments (100 µg), vehicle control (saline solution NaCl 0.9%), and positive control (budesonide 3 mg/kg, B7777, Sigma-Aldrich) were administered intranasally 60 min before each challenge (40 µL per mouse). Mice were sacrificed on day 25, and bronchoalveolar lavage (BAL) fluid and lungs were collected. Analyses of the total and differential cell counts in BAL fluid, as well as cytokine levels in lung homogenate, were conducted. The study was performed in accordance with the Artimmune/CNRS Orleans animal facility accreditation for experimentation (number F-45-234-006) and the asthma project accreditation for experimentation (CLE-CECCO-2014; ApaFis#25610).

### 2.24. Statistical Analysis

Statistical analyses and data representation were performed using GraphPad Prism 9, software version 9.4.1 (GraphPad). The normal distribution was assessed by applying the Shapiro–Wilk normality test. For multiple comparisons, unpaired one-way ANOVA followed by the Holm–Sidak post hoc test (when selecting a set of means to compare) or Dunnett’s post hoc test (when comparing every mean to a control mean) was performed. Unless otherwise indicated, data in graphs show the mean ± SD. *p* value of < 0.05 were deemed significant (*, *p* < 0.05; **, *p* < 0.001; ***, *p* < 0.0001).

## 3. Results

### 3.1. Stability Improvement of the ALCAM-Targeting Single-Chain Variable Fragment IF8

**Figure 1:**
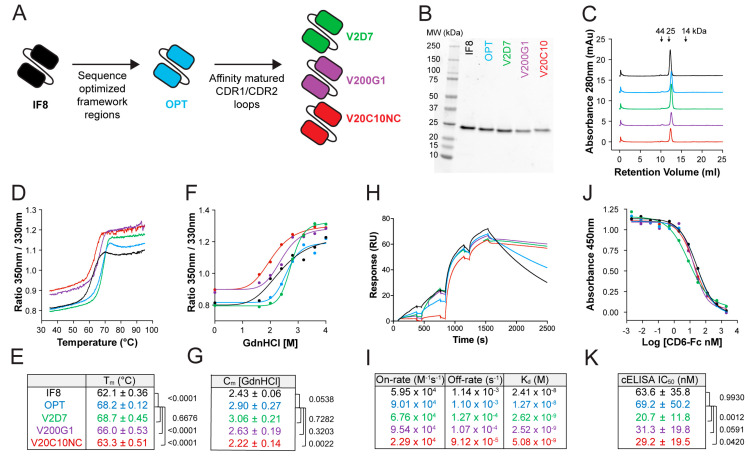
Framework and affinity improvements to IF8 yield superiorly stable, high-affinity anti-ALCAM scFv fragments. (**A**) Schematic representation of stability and affinity improvement steps performed in the generation of optimized scFv variants. (**B**–**K**) Side-by-side biophysical and functional comparison of the original (IF8), framework optimized (OPT), and affinity-matured (V2D7, V200G1, V20C10NC) purified scFv variants. Color coding is as shown in (**A**). (**B**) SDS-PAGE under nonreducing conditions. Expected molecular weight of the scFvs: 24 kDa. (**C**) FPLC profile on a Superdex 75 Increase column. (**D**) Representative plot of thermal unfolding curves as assessed by nanoDSF. (**E**) Quantification of T_m_ (mean ± SD of n = 3–4 independent measurements per variant). (**F**) Representative plot of protein unfolding in GdnHCl as assessed by nanoDSF. (**G**) Quantification of C_m_ (mean ± SD of n = 2–4 independent measurements per variant). (**H**) Representative plot of the binding of the scFv fragments to immobilized hALCAM as assessed by single-cycle SPR. Sequential injection of the antibodies at 30 nM, 60 nM, 120 nM, and 240 nM. (**I**) Quantification of the affinity constant (K_d_) on-rate and off-rate (mean from n = 2 independent runs). (**J**) Representative plot of the CD6-Fc competition ELISA. ScFv variants outcompete CD6-Fc for binding to hALCAM at increased concentrations, reducing the CD6-Fc absorbance readout. (**K**) Quantification of the IC_50_ from CD-Fc competition ELISA (mean ± SD of n = 4–14 independent experiments per variant). Statistics: One-way ANOVA, Holm–Sidak multiple comparison test (**E**,**G**,**K**).

Despite the important role of ALCAM in immune-mediated disorders, so far, only a few antibodies blocking mouse ALCAM in vivo have been described [7,10]. One particular clone that binds both human and murine ALCAM, the single-chain variable fragment IF8, was isolated from the naïve synthetic ETH-2 antibody phage library but was never extensively characterized [30]. We previously reformatted this clone as a bivalent scFv-Fc fusion protein (IF8-Fc) after appending the Fc fragment of murine IgG1 and demonstrated the in vivo efficacy of ALCAM blockade in a murine allograft rejection model [7]. While IF8-Fc worked well in our initial animal studies that involved a systemic route of administration, its size (>100 kDa) and format are suboptimal for use as a topical treatment. In this study, we first produced scFv IF8, confirmed its homogeneity by SEC and SDS-PAGE (Figure 1A–C), and determined its biophysical characteristics. The scFv IF8 was found to have moderate thermal and chemical stability with a T_m_ of 62.1 °C (Figure 1D,E) and a C_m_ of 2.43 M GdnHCl (Figure 1F,G). Moreover, scFv IF8 bound hALCAM with a mid-nanomolar affinity (K_d_ 24.1 nM; Figure 1H,I). In line with previous findings [30], scFv IF8 directly competed with its natural T cell-expressed ligand CD6 for binding to hALCAM with a half-maximal inhibitory concentration IC_50_ of 63.6 nM (Figure 1J,K). To further improve the stability of IF8, we grafted all CDRs and selected residues in the vicinity of the CDRs onto a framework with known stability [36,37] to yield the optimized scFv OPT (Figure 1A and Figure S1). Sequence profiling using the SAbPred Therapeutic Antibody Profiler [39], which compared the sequence of scFv OPT against five computational developability guidelines, confirmed a developability profile that was optimally aligned with that of >500 clinical-stage therapeutics (Figure S1B). In comparison to the original sequence of IF8, the sequence of OPT contained more favorable patches of positive charge (PPC) and patches of surface hydrophobicity (PSH) scores across the CDR vicinity (Figure S1B). In comparison to scFv IF8, scFv OPT had an increased T_m_ (increase of 6 °C; Figure 1D,E) and C_m_ (increase of 0.45 M GdnHCl; Figure 1F,G). In line with these biophysical improvements, production yields of scFv OPT were routinely 2–3 fold higher than those of scFv IF8. Importantly, scFv OPT retained a mid-nanomolar affinity for hALCAM (2-fold improved K_d_; Figure 1H,I) and directly competed with CD6 at levels equal to those of scFv IF8 (Figure 1J,K). These findings identified scFv OPT as a stability-improved scFv with an affinity for hALCAM equivalent to that of IF8.

### 3.2. Affinity Maturation of the ALCAM-Targeting scFv OPT

In a second step, we set out to improve the affinity of the stability-improved scFv OPT by affinity maturation using phage display (Figure S2). Considering that the synthetic phage display library (ETH-2) used for the isolation of the parent scFv IF8 was generated by the randomization of CDR3 residues of the V_L_ and V_H_ domains [49], we first randomized residues in the CDR1 loops of scFv OPT in both the V_L_ and V_H_ and selected the V2D7 clone by off-rate screening (Figure S2). In the next step, we randomized the CDR2 loops of scFv V2D7 and isolated the V200G1 and V20C10NC clones (Table 4, Figure 1A). Each selected clone was successfully produced in CHO cells, purified by protein A affinity chromatography, and recovered in a monomeric state to give a yield of >10 mg/mL with >95% purity (Figure 1B,C). In comparison to the parental scFv IF8, all three clones, i.e., V2D7, V200G1, and V20C10NC, displayed a 5–10-fold improved affinity for hALCAM (K_d_ 2.5–5 nM; Figure 1H,I) and performed significantly better in the CD6 competition ELISA (2- to 3-fold reduced IC_50_; Figure 1J,K). However, in the case of the V200G1 and V20C10NC clones, the improved affinity came at the expense of reduced thermal and chemical stability (Figure 1D–G). The remaining high-affinity clone V2D7 retained superior stability in comparison to the IF8 clone and was selected for additional formatting work. Notably, scFv V2D7 was highly cross-reactive and bound to human, mouse, rat, and cynomolgus monkey ALCAM in the single-digit nM range (Figure S3A–D).

### 3.3. Generation of Stable, High-Affinity ALCAM-Targeting Fragments of Various Formats

**Figure 2:**
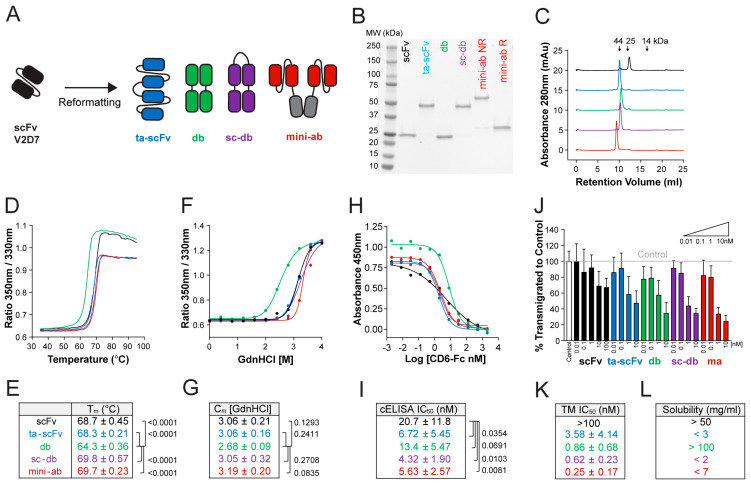
Bivalent formats of V2D7 retain stability and have improved functional activity. (**A**) Schematic representation of the antibody formats generated for screening. (**B**–**G**) Side-by-side biophysical and functional comparison of purified antibody formats (scFv, ta-scFv, db, sc-db, and mini-ab). Color coding is as shown in (**A**). (**B**) SDS-PAGE. Expected molecular weights: scFv: 24.2 kDa; ta-scFv: 49.7 kDa; db: 24.1 kDa; sc-db: 48.7 kDa; mini-ab: 59.6 kDa under nonreducing (NR) and 30 kDa under reducing (R) conditions. (**C**) FPLC profile on a Superdex 75 Increase column. (**D**) Representative plot of thermal unfolding curves as assessed by nanoDSF. (**E**) Quantification of T_m_ (mean ± SD of n = 2–5 independent measurements per variant). (**F**) Representative plot of protein unfolding in GdnHCl as assessed by nanoDSF. (**G**) Quantification of C_m_ (mean ± SD of n = 2–4 independent measurements per format). (**H**) Representative plot of the CD6-Fc competition ELISA. All variants outcompete CD6-Fc for binding to hALCAM at increased concentrations, reducing the CD6-Fc absorbance readout. (**I**) Quantification of the IC_50_ from the CD6-Fc competition ELISA (mean ± SD of n ≥ 3 independent experiments). (**J**) DC transmigration assay. Bivalent formats efficiently reduce DC transmigration across LEC monolayers in a dose-dependent manner. The plot shows mean ± SD from two pooled independent experiments with four replicates each. (**K**) Quantification of the IC_50_ from the DC transmigration assay depicted in (**J**). (**L**) Observed solubility of each format in PBS (pH 7.2–7.4). Statistics: One-way ANOVA, Holm–Sidak multiple comparison test (**E**,**G**,**I**).

In order to identify the best format for topical use, we reformatted scFv V2D7 into several small bivalent fragments, namely, ta-scFv, db, sc-db, and mini-ab (Figure 2A, Table 1). All formats were successfully produced and purified at yields of >10 mg/L and >95% purity, as assessed by FPLC (Figure 2B,C). Except for the db, bivalent formats of V2D7 had relatively similar T_m_ and C_m_ values to those of the scFv format (Figure 2D–G). As expected, in comparison to the scFv format, the bivalent formats of V2D7 were more efficient in outcompeting CD6 for binding to hALCAM (Figure 2H,I). In addition to characterizing the different antibody formats in our CD6 competition ELISA, we also investigated their ability to block ALCAM–ALCAM interactions in a cell-based assay. To this end, we established an in vitro DC transmigration assay across a lymphatic endothelial cell (LEC) monolayer. DCs and LECs both express ALCAM, and this migratory step is thought to primarily depend on homophilic ALCAM–ALCAM interactions [4,7]. Surprisingly, whereas bivalent formats of V2D7 effectively blocked DC transmigration across LEC monolayers at subnanomolar concentrations and in a dose-dependent manner, the scFv V2D7 only marginally blocked DC transmigration at concentrations as high as 100 nM (Figure 2J,K).

In addition to the stability and activity aspects, we also determined the solubility limits of the different V2D7 formats in PBS at pH 7.2–7.4. Fragments were concentrated by centrifugal filtration, and after overnight storage at RT, the concentration of the soluble fraction was measured (Figure 2L). The bivalent sc-db, ta-scFv, and mini-ab formats showed visible aggregation upon filtration with recorded solubility limits of less than 2, 3, and 7 mg/mL, respectively (Figure 2L). In contrast, the scFv and db formats showed no signs of aggregation upon filtration and remained soluble at concentrations of greater than 50 mg/mL and 100 mg/mL, respectively (Figure 2L). Additionally, a long-term stability study confirmed that db V2D7 and scFv V2D7 were stable at various temperatures (−80 °C, 4 °C, 22 °C, 37 °C) (Figure S3E–H) and that db V2D7 retained biological activity over 6 months (Figure S3I,J) (not assayed for the scFv).

### 3.4. Improvements in Stability and Affinity Are Similar between Antibody Clones in scFv and db Formats

**Figure 3:**
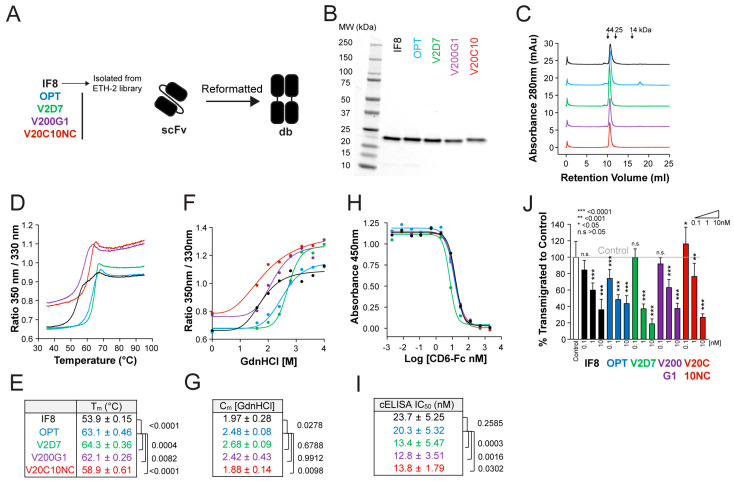
Diabody (db) formats of optimized anti-ALCAM scFv variants retain improved stability and activity. (**A**) Schematic representation of stability and affinity improvement steps performed in the generation of optimized db variants. (**B**–**G**) Side-by-side biophysical and functional comparison of the original (IF8), framework optimized (OPT), and affinity-matured (V2D7, V200G1, V20C10NC) purified db variants. Color coding is as shown in (**A**). (**B**) SDS-PAGE under nonreducing conditions. Expected molecular weight of dbs by SDS-PAGE (i.e., denatured, linearized): 24 kDa and by FPLC: 48 kDa (i.e., native protein). (**C**) FPLC profile on a Superdex 75 Increase column. (**D**) Representative plot of thermal unfolding curves as assessed by nanoDSF. (**E**) Quantification of T_m_ (mean ± SD of n = 5–6 independent measurements per variant). (**F**) Representative plot of protein unfolding in GdnHCl as assessed by nanoDSF. (**G**) Quantification of C_m_ (mean ± SD of n = 3–4 independent measurements per variant). (**H**) Representative plot of the CD6-Fc competition ELISA. Db variants outcompete CD6-Fc for binding to hALCAM at increased concentrations, reducing the CD6-Fc absorbance readout. (**I**) Quantification of the IC_50_ from the CD6-Fc competition ELISA (mean ± SD of n ≥ 5 independent experiments). (**J**) DC transmigration assay. Db variants reduce DC transmigration across LEC monolayers in a dose-dependent manner. The plot shows the mean ± SD from two pooled independent experiments with four replicates each. The statistical comparison versus control (gray line) is shown. Statistics: One-way ANOVA, Holm–Sidak multiple comparison test (**E**,**G**,**I**) or Dunnett’s multiple comparison test (**J**).

To investigate whether the improvements in stability and affinity observed in the scFv format (Figure 1) would also remain apparent in the bivalent format, we produced the different clones (IF8, OPT, V2D7, V200G1, V20C10NC) in db format (Figure 3A). All db clones were successfully produced and purified at yields > 10 mg/L with >95% purity (Figure 3B,C). Although the T_m_ and C_m_ of all clones in the db format were consistently lower compared to those previously measured for the corresponding scFv format, the relative differences between the clones were grossly comparable (Figure 3E,G). For example, the T_m_ and C_m_ of OPT in db format were again significantly improved compared to the values measured for IF8 in db format, whereas of the affinity-matured clones, only V2D7, but not V200G1 or V20C10NC, retained superior thermal and chemical stability in the db format (Figure 1E,G and Figure 3E,G). All db clones directly competed with CD6, with a significant improvement observed for the affinity-matured clones V2D7, V200G1, and V20C10NC (Figure 3H,I). Moreover, all affinity-matured clones effectively blocked DC transmigration across LEC monolayers, although no clear improvement compared to IF8 was observed in this case, possibly due to the variability of the assay (Figure 3J).

### 3.5. Smaller Antibodies Penetrate Better through In Vitro 3D Human Corneal Epithelium

**Figure 4:**
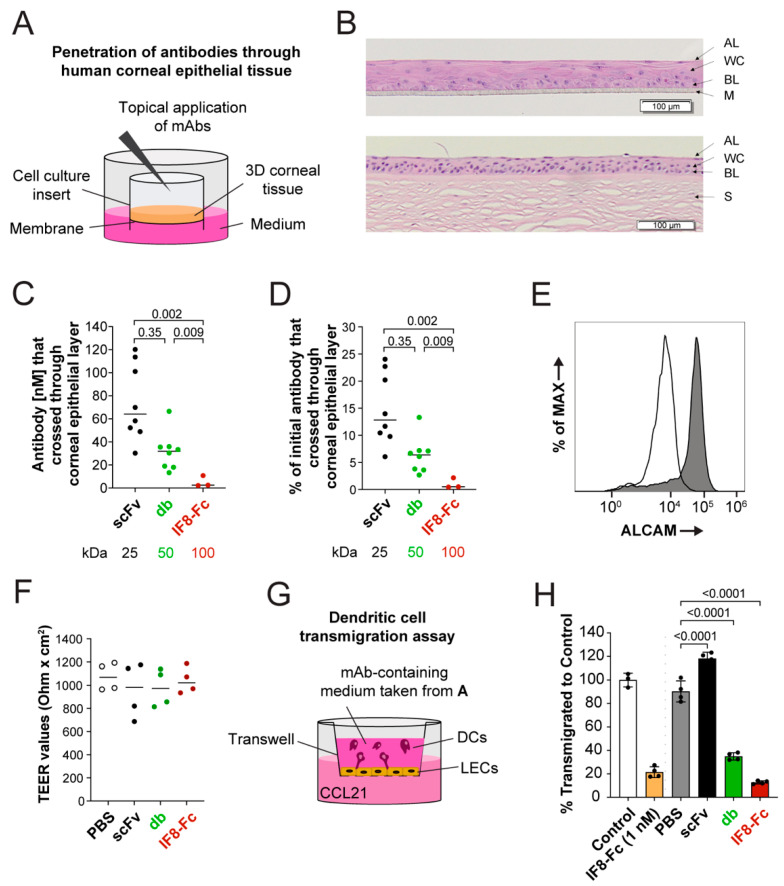
Smaller antibody formats more effectively penetrate the human corneal epithelium and remain functional following penetration. (**A**) Schematic depiction of the 3D human epithelial corneal tissue model grown in cell culture inserts. IF8-Fc, db V2D7, and scFv V2D7 were applied topically on the corneal tissue layer at a concentration of 500 nM and left to penetrate for 6 h. (**B**) H&E-stained cross-section of 3D human corneal epithelial tissue model (upper image) and human corneal tissue (bottom image). AL: apical layer; WC: wing cells; BL: basal layer; M: microporous membrane; S: stroma (**C**) Percentage (%) of the antibody compared to the initial input or (**D**) antibody concentration (nM) that crossed the corneal epithelial layer after 6 h determined with an AlphaLISA assay. Representative plots of one out of two experiments performed. Mean of n = 3–8 replicates. (**E**) Human corneal epithelial cells express ALCAM, as assessed by flow cytometry (n = 1). (**F**) Transepithelial electrical resistance (TEER) of the 3D corneal tissue model measured after the penetration assay. Representative plot of one out of two experiments performed. Mean of n = 4 replicates. (**G**) Schematic depiction of the dendritic cell (DC) transmigration assay performed in transwells containing a lymphatic endothelial cell (LEC) monolayer. The medium from the penetration assay containing mAbs that crossed the corneal epithelial layer was applied to the top of the transwell to assess its ability to block DC transmigration towards the chemokine CCL21. (**H**) DC transmigration performed in the presence of the medium collected from the corneal tissue penetration assays (see **A**–**D**) with scFv, db and IF8-Fc (one technical replicate out of two shown) or negative control PBS (n = four transwells per condition). IF8-Fc at 1 nM was used as a positive control, and PBS was used as the negative control (control). Representative plot of one out of two experiments. Statistics: One-way ANOVA, Holm–Sidak multiple comparison test (**C**,**D**) or Dunnett’s multiple comparison test (**H**).

Previous studies have suggested that, in comparison to full-length antibodies, smaller antibody fragments penetrate epithelial barriers and access target tissues more easily [21,22,24]. To determine whether this is the case for our anti-ALCAM fragments, we investigated the penetration of scFv V2D7, db V2D7, and IF8-Fc (which substantially differ in terms of molecular weight, stability, and in vitro functional activity (Table 5)) across an in vitro reconstructed 3D human corneal epithelium (Figure 4A). Specifically, we used a 3D human corneal tissue model that represents an in vitro reconstructed corneal tissue formed by multilayered human corneal epithelial cells (4–5 layers, Figure 4B). The latter possesses a similar tissue structure (Figure 4B), barrier properties, and expression of cornea-specific markers compared to the in vivo human cornea [50,51]. Fragments were applied at a concentration of 500 nM (corresponding to IF8-Fc 50 mg/mL, db 24 mg/mL, scFv 12 mg/mL) to the upper side of the corneal tissue, and after six hours, the concentration of antibodies that had penetrated through the corneal tissue into the lower media-containing chamber was determined by AlphaLISA (Figure 4A). As expected, the level of each of the fragments detected in the lower chamber displayed strong size-dependence and was significantly reduced for IF8-Fc in comparison to the smaller fragments (Figure 4C,D). In line with the expression of ALCAM by various epithelial cells [52,53,54,55], we also detected ALCAM expression on corneal epithelial cells by flow cytometry (Figure 4E). However, TEER measurements confirmed that the addition of the anti-ALCAM antibodies did not affect the epithelial barrier integrity, despite binding to corneal epithelial cells (Figure 4F). These findings confirmed the penetration of anti-ALCAM fragments across an epithelial barrier in a size-dependent manner. In order to determine whether the fragments remained functional after penetration, medium from the lower chamber containing the penetrated antibody fragments was collected, and its blocking activity was evaluated in a DC transmigration assay (Figure 4G). In comparison to the lower chamber solution of the vehicle control (PBS), replicate solutions of V2D7 db and IF8-Fc effectively blocked DC transmigration across LEC monolayers (Figure 4H). Similarly to what was observed in Figure 2J,K, the scFv fragment was ineffective for blocking DC transmigration across an LEC monolayer in this assay (Figure 4H). Taken together, these findings demonstrate a clear size-dependence in the antibody fragments’ ability to penetrate the corneal epithelium and further indicate that the antibodies retained their activity upon penetration across an epithelial barrier.

### 3.6. Topical Treatment with IF8-Fc and scFv V2D7 Reduces Immune Cell Infiltration in a Mouse Model of Allergic Asthma

**Figure 5:**
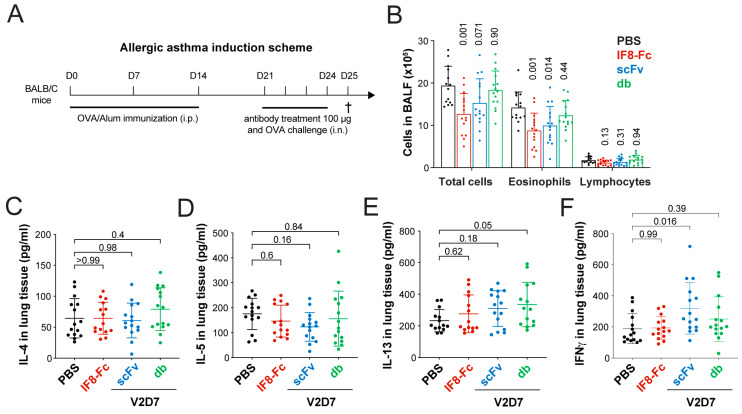
Intranasal treatment with anti-ALCAM antibody fragments reduces immune cell infiltration in a mouse model of asthma. (**A**) Schematic representation of the ovalbumin (OVA)-induced allergic asthma mouse model. Mice were treated intranasally with vehicle (PBS), IF8-Fc, V2D7 scFv, or V2D7 db 60 min prior to OVA challenges (100 µg dose) on days 21, 22, 23, and 24. On day 25, mice were sacrificed, and the (**B**) number of immune cells in the bronchoalveolar lavage (BAL) fluid and (**C**–**F**) concentrations of IL-4, IL-5, IL-13, and IFNγ in lung tissue homogenates were determined. Data are from a single study with 15 animals per group. The mean ± SD are shown. Statistics: One-way ANOVA, Dunnett’s multiple comparison test (**B**–**F**).

ALCAM has previously been brought forward as a target for allergic asthma. Specifically, in an OVA-induced murine asthma model, intranasal treatment with a polyclonal rabbit anti-ALCAM antibody at the time of OVA challenge reduced allergic symptoms, such as the leukocyte count and Th2 cytokine (i.e., IL-4, IL-5, and IL-13) levels in BAL fluid [10]. The study further suggested that, in this model, the main impact of ALCAM blockade comes from blocking CD6-induced T cell activation and proliferation [10]. Considering that the latter study demonstrated the therapeutic impact of ALCAM blockade using a suboptimal (likely immunogenic) polyclonal antibody preparation, we next investigated whether intranasal treatment with the parental antibody IF8-Fc or with our newly generated, stability- and affinity-improved anti-ALCAM fragments would also be effective for preventing allergic inflammation in the lungs. We tested the db V2D7, but also the monovalent scFv V2D7, which displayed the greatest transepithelial penetration (Figure 4C,D) with high solubility (Figure 2L) and, at the same time, avidly blocked CD6 binding in the competition assay (Figure 2H,I). BALB/c female mice were immunized to OVA on days 0, 7, and 14 and challenged daily from days 21 to 24 with OVA applied intranasally (Figure 5A). Test compounds (IF8-Fc, db V2D7, and scFv V2D7) or the corticosteroid control treatment (budesonide) were administered intranasally 60 min before each challenge at a weight-equivalent dose of 100 µg/mouse. On day 25, the severity of allergic asthma was determined by analyzing the cell counts in the BAL fluid (Figure 5B) and the cytokine levels in lung homogenates (Figure 5C–F). Antibody treatment did not impact mouse weight, whereas budesonide reduced the weight by approximately 10% (Figure S4A). In comparison to uninduced control mice, mice challenged with OVA showed an increased number of total immune cells, which mainly consisted of eosinophils in the BAL fluid (Figure S4B) and elevated levels of the inflammatory cytokines IL-4, IL-5, and IL-13 in lung homogenates (Figure S4C–F), confirming the induction of an allergic response. Corticosteroid treatment (budesonide) effectively reduced eosinophilic and lymphocytic cell counts in BAL fluid (−92.3% and −34.7%, respectively, Figure S4B) as well as IL-4 and IL-5 levels in lung homogenates (−70.5% and −69.5%, respectively, Figure S4C–F). Similar to a previous report on the administration of polyclonal anti-ALCAM antibodies [10], intranasal treatment with IF8-Fc significantly reduced eosinophilic cell counts in BAL fluid by approximately 38% (Figure 5B). However, IF8-Fc had no effects on IL-4, IL-5, IL-13, or IFNγ levels in lung homogenates (Figure 5C–F). Additionally, scFv V2D7 significantly reduced the eosinophilic cell count (−30%) but had no effects on the IL-4, IL-5, and IL-13 levels (Figure 5B–E). In contrast, V2D7 in db format did not display any in vivo activity (Figure 5B–F). Taken together, these findings confirm the in vivo therapeutic activity of the ALCAM-blocking parental antibody construct IF8-Fc and the affinity-matured and stability-optimized clone V2D7 in scFv format in a mouse model of asthma.

## 4. Discussion

ALCAM has been identified as a potential therapeutic target for several immune-mediated diseases, including corneal graft rejection, asthma, atopic dermatitis, food allergy, and multiple sclerosis [7,10,14,15]. While the broad expression pattern of ALCAM makes it a less attractive target for systemic administration, topical application could represent a promising alternative for targeting ALCAM with mAbs in surface-exposed tissues, such as the lungs or the cornea. The latter would require ALCAM-targeting mAb fragments with high stability, solubility, and affinity, which we generated and characterized in this study.

Several approaches have been used to engineer and improve the intrinsic stability of mAb fragments (reviewed in [56,57,58]). In this study, a combination of CDR grafting and rational design [36,37] was applied to improve the stability of a previously isolated anti-ALCAM scFv (IF8) [30]. The fact that the sequence of the optimized scFv OPT achieved more favorable PPC and PSH scores across the CDR vicinity in comparison to the starting sequence of scFv IF8 (Figure S1B) likely explains the associated stability improvements of the OPT fragment (Figure 1E,G). Indeed, unfavorable PPC scores have been linked with poorer biophysical properties and a propensity for aggregation [39]. Except for the db format, thermal and chemical stability was generally conserved across the different stability-improved OPT-based antibody formats (Figure 2D–G). Conversely, each of the formats had vastly different solubility limits in PBS (pH 7.4) (Figure 2L). The strikingly higher solubility of the scFv and db formats (>50 mg/mL) suggests that this variation might be dependent on the simplicity of these formats, which both contain one single interdomain linker. Both linker sequence and domain orientation have been shown to impact the biophysical properties of antibody fragments [59,60]. In the case of ta-scFv V2D7, changing the length or sequence of the linker or switching the domain orientation failed to improve the solubility. Most interestingly, despite the sc-db V2D7 and db V2D7 having the same molecular arrangement, their solubility limits differed drastically. This suggests the occurrence of oligomeric aggregation mediated by interchain V_H_–V_L_ interactions. Similar findings have been reported for a αCEA × αCD3 bispecific T cell engager construct, in which the ta-scFv format displayed a greater tendency to aggregate as compared to the two-chain diabody [61,62].

We used two in vitro assays to characterize the activity of our antibodies, namely, a competition assay investigating the antibodies’ ability to block CD6–ALCAM interactions, which are important for T cell activation [3,7,10], as well as a cell-based assay investigating the antibodies’ ability to block DC transmigration across the lymphatic endothelium, which is dependent on ALCAM–ALCAM interactions [4,7]. Screening the different clones and formats in these functional assays revealed that the performance of the anti-ALCAM fragments in the in vitro DC transmigration assay highly depended on the antibody valency (Figure 2J,K), whereas the valency dependence was much less pronounced in the CD6 competition assay (Figure 2H,I). In the DC transmigration assay, anti-ALCAM fragments interfered with the migration of DCs by blocking the interaction of DC-expressed ALCAM with LEC-expressed ALCAM. These ALCAM–ALCAM interactions are formed in *trans* upon binding of ALCAM’s membrane-distal immunoglobulin domains and further stabilized by clustering-induced *cis* interactions between membrane-proximal immunoglobulin domains of ALCAM molecules on the cell’s surface [1]. It is perceivable that the monovalent format of the scFv does not suffice to compete with the avidity and clustering effects of ALCAM–ALCAM interactions. Conversely, the scFv was quite effective compared to its bivalent counterparts in the CD6 competition ELISA (only 1.5- to 4-fold higher IC_50_, Figure 2I). Amongst the bivalent formats, the ta-scFv, sc-db, and mini-ab were equally efficient in displacing CD6, whereas the IC_50_ of the db format was approximately 2- to 3-fold higher (Figure 2I). Considering the opposing orientation of the binding sites in the db and the rigidity of the format due to the short linker sequence, it is possible that, frequently, only one single V_H_–V_L_ pair managed to bind to ALCAM in the setup of our CD6 competition assay. On the other hand, in the DC transmigration assay, where ALCAM molecules were likely to be much more densely clustered on the cell surface, the bivalent antibody formats displayed a strong avidity effect, translating into strong blocking activity.

Intranasal treatment with a polyclonal anti-ALCAM antibody was previously shown to reduce inflammatory symptoms in an OVA-induced model of allergic asthma [10]. In accordance with this study, treatment with both IF8-Fc and scFv V2D7 significantly reduced eosinophilic cell numbers in BAL fluid (Figure 5). The fact that the cortisone (budesonide) control performed so well (Figure S4) is not surprising, since cortisone is known to be highly efficacious in this mouse model [63]. However, since a considerable number of patients are insensitive to cortisone treatment [64,65], and corticosteroids alone typically do not suffice for the management of severe asthma [66,67], there remains a strong need for the development of further drugs targeting other pathways, such as cytokines, cytokine receptors, or cell adhesion molecules such as CD6 or ALCAM [68,69].

Surprisingly, db V2D7 did not display any clear activity (*p* = 0.44) upon intranasal delivery, despite performing slightly better than scFv V2D7 in the in vitro CD6 competition assay (Figure 2H,I) and clearly better in the DC transmigration assay (Figure 2J,K). Overall, the discrepancy in performance between scFv V2D7 and db V2D7 could be due to several reasons: differences in either the molar concentrations applied during equimass treatment (i.e., two times more scFv molecules compared to db), in the antibodies’ in vivo stability, as suggested from their biophysical characterization (Table 5), or in the antibodies’ ability to penetrate the nasal mucosa and move across the epithelial border. Notably, compared to IF8-Fc, scFv V2D7 only marginally reduced DC transmigration in vitro, whereas both fragments blocked CD6–ALCAM interactions (Table 5). This indicates that, as previously suggested [10], the reduced T cell activation and proliferation is the critical mechanism by which ALCAM blockade reduces inflammatory symptoms in OVA-induced asthma. Interestingly, the neonatal Fc receptor (FcRN) has been suggested to support the transport of Fc-containing antibodies across the nasal epithelium [19,70,71]. Fc-mediated active transepithelial uptake might therefore have additionally enhanced the in vivo activity of intranasally administered IF8-Fc and compensated for the fact that IF8-Fc was administered at a four times lower molar concentration during equimass treatment compared to scFv V2D7. Further studies are needed to assess the suitability of anti-ALCAM mAbs upon delivery by inhalation, i.e., the most suitable route of topical administration for therapeutic proteins in human asthma [72]. At this point, our study confirms ALCAM as a therapeutic target for asthma and demonstrates the in vivo activity of both IF8-Fc and scFv V2D7 upon intranasal administration.

In contrast to the lungs, where the topical administration of therapeutic proteins typically involves aerosolization, antibody fragments are administered in aqueous solutions (i.e., eye drops) for the treatment of corneal disease. However, in comparison to the thin one-cell-layered lung epithelium, the corneal epithelium is composed of multiple cell layers, forming a barrier that is much harder to penetrate for protein-based therapeutics. Our assays performed in an in vitro 3D human corneal epithelium model revealed a clear superiority of smaller antibody fragments in penetrating the epithelium. Few studies have compared the scFv and IgG formats [21,24], and only one other study included a third format (mini-ab) in the comparison [22]. These experiments were typically performed ex vivo in mouse, rabbit, or cat eyes and yielded similar results to ours, namely, a size-dependent penetration of antibody fragments across the corneal epithelium. Therefore, our results confirm that small and stable antibody fragments, such as the ones described in this study, could be promising for use as a topical, antibody-based eye drop treatment for inflammatory eye disease, avoiding the burden of systemic or intra-ocular injections whilst allowing the achievement of therapeutic concentrations in the eye.

## 5. Conclusions

In this study, we reported the generation and in vitro and in vivo functional characterization of a set of stability- and affinity-improved ALCAM-targeting mAb fragments. In comparison to the parent mAb IF8, the optimized mAb fragments were more efficacious in vitro in competing with CD6 for ALCAM binding or blocking DC transmigration across LEC monolayers. Moreover, using a 3D human epithelial corneal tissue model, we confirmed that smaller ALCAM-targeting mAb fragments displayed superior transepithelial penetration. Finally, we demonstrated the in vivo activity of one optimized mAb fragment upon topical application in a murine asthma model.

Overall, our findings suggest that the development of a topical and therefore more localized treatment with highly stable anti-ALCAM mAb fragments, as depicted in this study, could represent an alternative possibility for targeting the ALCAM–CD6 pathway and potentially treating certain immune-mediated disorders.

## Figures and Tables

**Table 1:** Linker sequences used to connect the variable heavy chain (V_H_) and the variable light chain (V_L_) domains in the different antibody fragments generated.

scFv	VH—GGGGSGGGGS *—VL
db	VH—GGSGG—VL
sc-db	VH—GGSGG—VL—GGGGSGGGGSGGGGS—VH—GGSGG—VL
ta-scFv	VH—GGGGSGGGGSGGGG—VL—GGGGSGGGGS—VH—GGGGSGGGGSGGGG—VL
mini-ab	VH—GGGGSGGGGSGGGG—VL—TPLGDTTHTSG—RMKQLEDKVEELLSKNYHLENEVARLKKLVGERGGCGG

* Note: IF8 scFv contains a (G_4_S)_3_ linker, whereas the other clones contain a (G_4_S)_2_ linker.

**Table 2:** Affinity-maturation library primers.

CDR1 affinity-maturation library primers		
a	LMB3long	5′—CAG GAA ACA GCT ATG ACC ATG ATT AC—3′
b	DP47CDR1rev	5′—AGC CTG GCG GAC CCA GCT CAT MNN MNN MNN GCT AAA GGT GAA TCC AGA GGC TGC—3′
c	DP47CDR1for	5′—GAG CTG GGT CCG CCA GGC TCC—3′
d	DPL16CDR1rev	5′—TCC TGG CTT CTG CTG GTA CCA GCT TGC MNN MNN MNN TCT GAG GCT GTC TCC TTG—3′
e	DPL16CDR1for	5′—TGG TAC CAG CAG AAG CCA GGA—3′
f	Fdseqlong	5′—GAC GTT AGT AAA TGA ATT TTC TGT ATG AGG– 3′
CDR2 affinity-maturation library primers		
a	LMB3long	5′—CAG GAA ACA GCT ATG ACC ATG ATT AC—3′
b	DP47CDR2rev	5′—GCC CTT CAC GGA GTC TGC GTA GTA TGT MNN ACC ACC MNN MNN MNN AAT AGC TGA GAC CCA CTC C— 3′
c	DP47CDR2for	5′—ACA TAC TAC GCA GAC TCC GTG AAG GGC—3′
d	DPL16CDR2revOpt	5′—TTC TGG GAT CCC TGA GGG CCG MNN MNN TTT MNN ATA CAC GAC AAG TAC AGG GGC C—3′
e	DPL16CDR2forOpt	5′—CGG CCC TCA GGG ATC CCA GAA—3′
f	Fdseqlong	5′—GAC GTT AGT AAA TGA ATT TTC TGT ATG AGG—3′

M and N are defined according to IUPAC nomenclature (M = A/C, N = A/T/G/C).

**Table 3:** Strategy for affinity maturation using phage display.

Affinity maturation of CDR1 of the OPT scFv clone			
	Biotinylated hALCAM V1	Competition	Comment
Round 1	500 nM	n.a.	n.a.
Round 2	50 nM	200 nM Opt scFv	Led to V2D7 clone
Affinity maturation of CDR2 of the V2D7 scFv clone			
Round 1	5 nM	n.a.	n.a.
Round 2a	5 nM	20 nM V2D7 scFv	Led to V20C10NC clone
Round 2b	5 nM	200 nM V2D7 scFv	Led to V200G1 clone

**Table 4:** CDR sequences of the affinity-matured clones.

	Clone	CDR1	CDR2	CDR3
Heavy chain (DP47)	IF8	SYAMS	AISGSGGSTYYADSVKG	GYVAFDY
	Opt	SYAMS	AISGSGGSTYYADSVKG	GYVAFDY
	V2D7	STGAMS	AISGSGGSTYYADSVKG	GYVAFDY
	V20C10NC	STGAMS	AISG*TGGTTYYADSVKG	GYVAFDY
	V200G1	STGAMS	AISGSGGSTYYADSVKG	GYVAFDY
Light chain (DPL16)	IF8	QGDSLRSYYAS	GKNNRPS	NSSPPFSAEVV
	Opt	QGDSLRSYYAS	GKNNRPS	NSSPPFSAEVV
	V2D7	QGDSLRSGYAS	GKNNRPS	NSSPPFSAEVV
	V20C10NC	QGDSLRSGYAS	PKM * SRPS	NSSPPFSAEVV
	V200G1	QGDSLRSGYAS	GKTGRPS	NSSPPFSAEVV

* Positively charged histidine residues in the CDR2 of the V20C10 clone were mutated to uncharged threonine and serine residues, respectively, generating V20C10NC.

**Table 5:** Biophysical and functional characterization of IF8-Fc, scFv V2D7, and db V2D7.

		Biophysical		Functional	
	MW (kDa)	Thermal Stability T_m_ (°C)	Chemical Stability C_m_ (M GdnHCl)	cELISA IC_50_ (nM)	DC TM IC_50_ (nM)
IF8-Fc	100	64 ± 0.66	3.06 ± 0.13	7.33 ± 3.91	0.44 ± 0.39
scFv V2D7	24	68.7 ± 0.45	3.06 ± 0.21	20.7 ± 11.8	>100
db V2D7	48	64.3 ± 0.36	2.68 ± 0.09	13.4 ± 5.47	0.86 ± 0.68

## Data Availability

The data presented in this study are available on request from the corresponding authors.

## Supplementary Materials

The following supporting information can be downloaded at: https://www.mdpi.com/article/10.3390/pharmaceutics15071841/s1, Figure S1: Stabilization of IF8 scFv by CDR grafting and framework optimization; Figure S2: Affinity maturation and phage display screening strategy performed for the identification of clone V2D7; Figure S3: Analysis of species cross-reactivity, stability, and functionality of clone V2D7 displays over time; Figure S4: Effects of intranasal treatment with anti-ALCAM antibody fragments or budesonide on immune cell infiltration in a mouse model of asthma.
