# Supplementary material for: Optimization and Characterization of Novel ALCAM-Targeting Antibody Fragments for Transepithelial Delivery
Source: Pharmaceutics. 2023 Jun 27;15(7):1841. doi: 10.3390/pharmaceutics15071841 (PMC10385607; doi:10.3390/pharmaceutics15071841)
Supplement: Supplementary file 1 [file pharmaceutics-15-01841-s001.zip › pharmaceutics-2414053-supplementary.pdf]

Supplementary figures

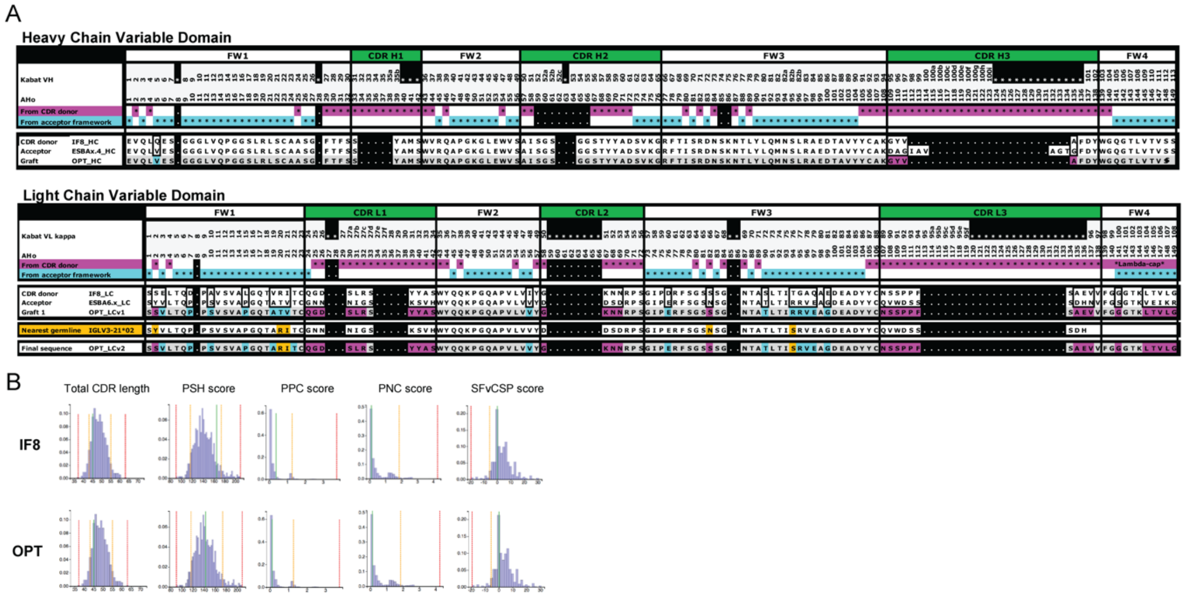

Figure S1: Stabilization of IF8 scFv by CDR grafting and framework optimization.

(A) Complementarity determining regions (CDRs) and important framework (FW) residues of IF8 (donor) were grafted onto stabilized frameworks (acceptor) to generate a stabilized graft (OPT). Mismatched residues between donor and acceptor frameworks are outlined in black. Donor and acceptor residues retained in the resulting graft are highlighted in purple and cyan, respectively. Residues conserved between donor and acceptor frameworks are highlighted in gray. In the case of the V_L, the resulting graft was further mutated to that of the nearest germline sequence (IGLV3-21*02). Mismatched germline residues incorporated into the final light chain sequence are highlighted in orange. Kabat and Honneger's numbering scheme (AHo) of amino acid residues is shown. **(B)** SAbPred Therapeutic Antibody Profiler (TAP) output comparing IF8 and OPT amino acid sequences against developability guidelines derived from >500 clinical-stage therapeutics. PSH: Patches of Surface Hydrophobicity across the CDR vicinity. PPC: Patches of Positive Charge across the CDR vicinity. PNC: Patches of Negative Charge across the CDR vicinity. SFvCSP: Structural Fv Charge Symmetry Parameter. Blue histograms show the distribution of clinical-stage therapeutics. Orange and red dashed lines show the score of amber flag (i.e., antibodies that lie within the 5% extremes of the distribution) and red flag (a previously unobserved value for that property) boundaries (as defined by [39]). Solid green lines indicate the scores of either IF8 or OPT (shown in the upper and lower rows, respectively).

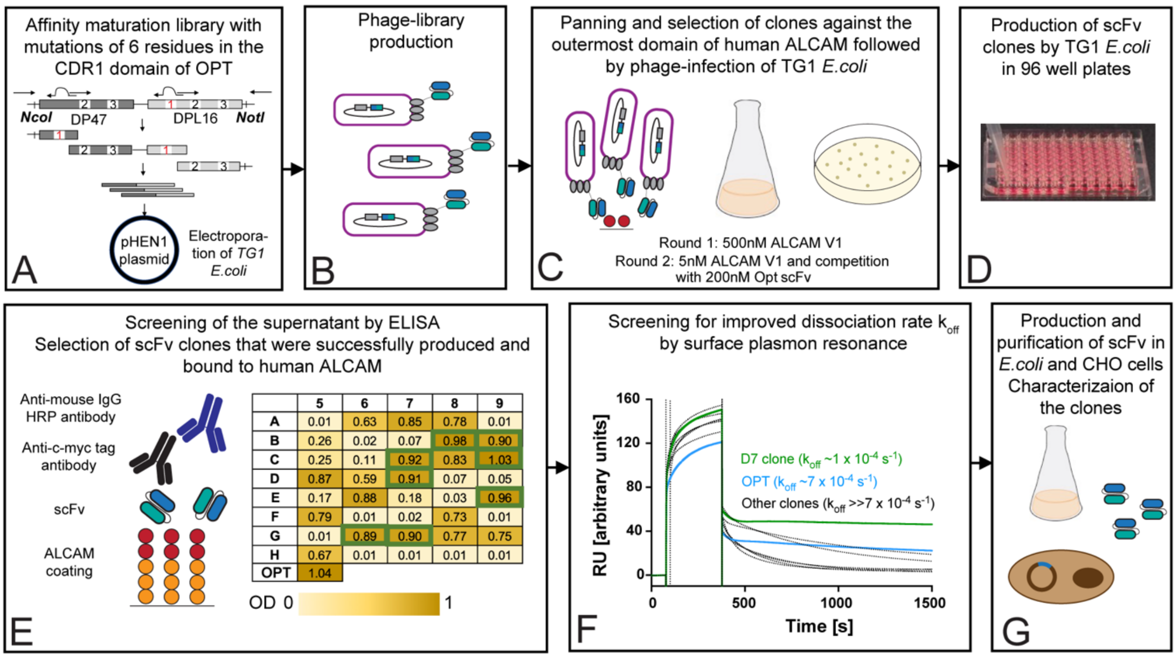

	5	6	7	8	9
A	0.01	0.63	0.85	0.78	0.01
B	0.26	0.02	0.07	0.98	0.90
C	0.25	0.11	0.92	0.83	1.03
D	0.87	0.59	0.91	0.07	0.05
E	0.17	0.88	0.18	0.03	0.96
F	0.79	0.01	0.02	0.73	0.01
G	0.01	0.89	0.90	0.77	0.75
H	0.67	0.01	0.01	0.01	0.01
OPT	1.04				

Figure S2: Affinity maturation and phage-display screening strategy performed for the identification of clone V2D7.

(**A-G**) Schematic representations of the affinity maturation and phage-display screening strategy. In **(E-F)**, representative data resulting in the discovery of the V2D7 clone are shown. **(A)** First, an affinity maturation library of the CDR1 domain from the OPT clone, containing mutations of three residues in the heavy chain DP47 and three residues in the light chain DPL16, was created. Subsequently, the library was cloned in the pHEN1 plasmid and electroporated in TG1 *E. coli*. **(B)** A phage-library was produced, and phages displayed the various scFv clones fused to the phage-derived coat protein pIII. **(C)** Phage-display selection was performed in two rounds of panning against the outermost domain (V1) of human ALCAM. The first round was performed against 500 nM ALCAM V1 and the second round was performed against 5 nM ALCAM V1 in the presence of the competitor OPT scFv. Binding phages were eluted and used to infect TG1 *E.coli*. Subsequently, the bacteria were plated on agar plates. **(D)** After phage-display selection, single colonies were picked from the agar plates and inoculated in 96-well plates containing bacterial growth media and left to produce scFv overnight. **(E)** The supernatant containing scFv clones was screened using ELISA. A 96-well plate was coated with human ALCAM, and successfully bound scFv clones were detected via their c-myc tag and the signal amplified via an HRP-coupled secondary antibody. Clones generating the highest optical density (OD) on the ELISA were selected for the further steps (bold green box). The ELISA results of the selection from round 2 leading to the discovery of the clone V2D7 (well D7) are shown. **(F)** The supernatant of the selected clones was screened by surface plasmon resonance for an improved dissociation rate k_{off} and compared to the k_{off} of OPT. **(G)** The most promising scFv clones in terms of the dissociation rate were produced and purified first from *E. coli*, followed by CHO cells, and then characterized.

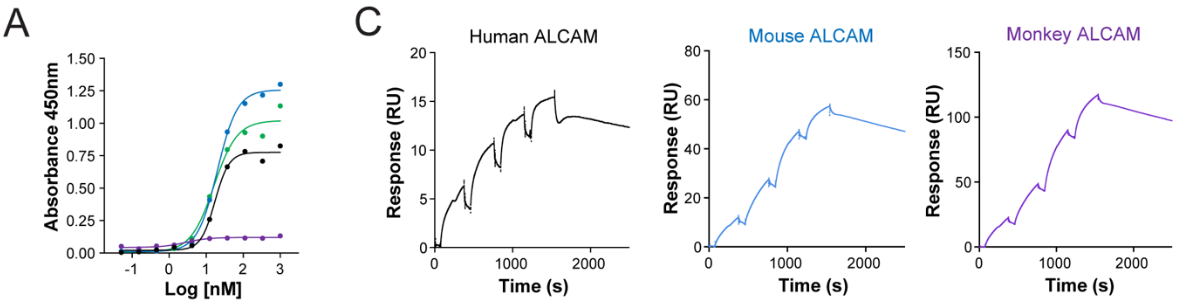

B

	V1 Identity (%)	ELISA EC_{50} (nM)
Human	Reference	18.2 ± 3.73
Mouse	94.6	25.4 ± 10.5
Rat	94.6	23.5 ± 8.98
Monkey	97.8	Inconclusive

D

	On-rate ($M^{-1}s^{-1}$)	Off-rate (s^{-1})	K_d (M)
Human	6.76E+04	1.27E-04	2.62E-09
Mouse	1.72E+04	1.50E-04	8.74E-09
Rat	n.d.	n.d.	n.d.
Monkey	3.75E+04	1.82E-04	4.86E-09

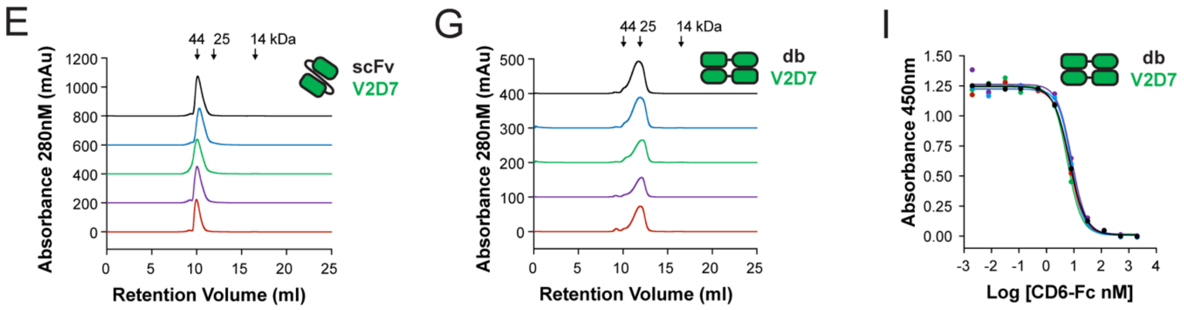

F

Diabody	Monomer content (%)		
Storage (days)	1	90	180
Control	96.5		
4°C	n.d.	95.9	96.7
22°C	n.d.	96.0	98.0
-80°C	n.d.	n.d.	95.3
37°C	96.4	n.d.	n.d.

H

ScFv	Monomer content (%)		
Storage (days)	1-7	90	180
Control	98.6		
4°C	n.d.	83.5	98.6
22°C	n.d.	98.6	98.4
-80°C	n.d	n.d.	98.3
37°C	95.9	n.d.	n.d.

J

cELISA IC_{50} (nM) 180 days
7.49 ± 0.68
7.45 ± 0.63
6.93 ± 1.72
7.63 ± 1.22
8.48 ± 0.26

Figure S3: Analysis of the species cross-reactivity, stability, and functionality of clone V2D7 over time.

(A) Species cross-reactive binding of V2D7 scFv to immobilized human, mouse, rat, and monkey ALCAM by direct ELISA (one representative plot). **(B)** Table showing the percentage identity of human ALCAM V1 to mouse, rat, and monkey ALCAM and quantification of IC_{50} from direct ELISA in (A) (mean ± SD of three independent experiments). **(C)** Species cross-reactive binding of V2D7 scFv to immobilized human, mouse, and monkey ALCAM by single-cycle surface plasmon resonance analysis. **(D)** Quantification of affinity (K_d), on-rate and off-rate (value from one side-by-side experimental run). **(E-H)** Stability of db V2D7 (60 mg/ml in PBS, pH 7.4, **E/F**) and scFv V2D7 (20 mg/ml in PBS, pH 7.4, **G/H**) at different storage temperatures (-80 °C, 4 °C, 22 °C, 37 °C) over time (1-7 days, 3 and 6 months), as assessed by FPLC analysis (plots in E and G are from the 6 month timepoint or from 1-7 days for the 37 °C condition). The monomeric content is shown in tables F and H as a percentage (n.d. not determined). Values from one experimental run. **(I)** Representative plot of the CD6-Fc competition ELISA performed with the db V2D7 stored for 6 months at different storage temperatures (-80 °C, 4 °C, 22 °C, 37 °C). **(J)** Quantification of IC_{50} from the CD6-Fc competition ELISA in (I) (mean ± SD of n=2 independent experiments).

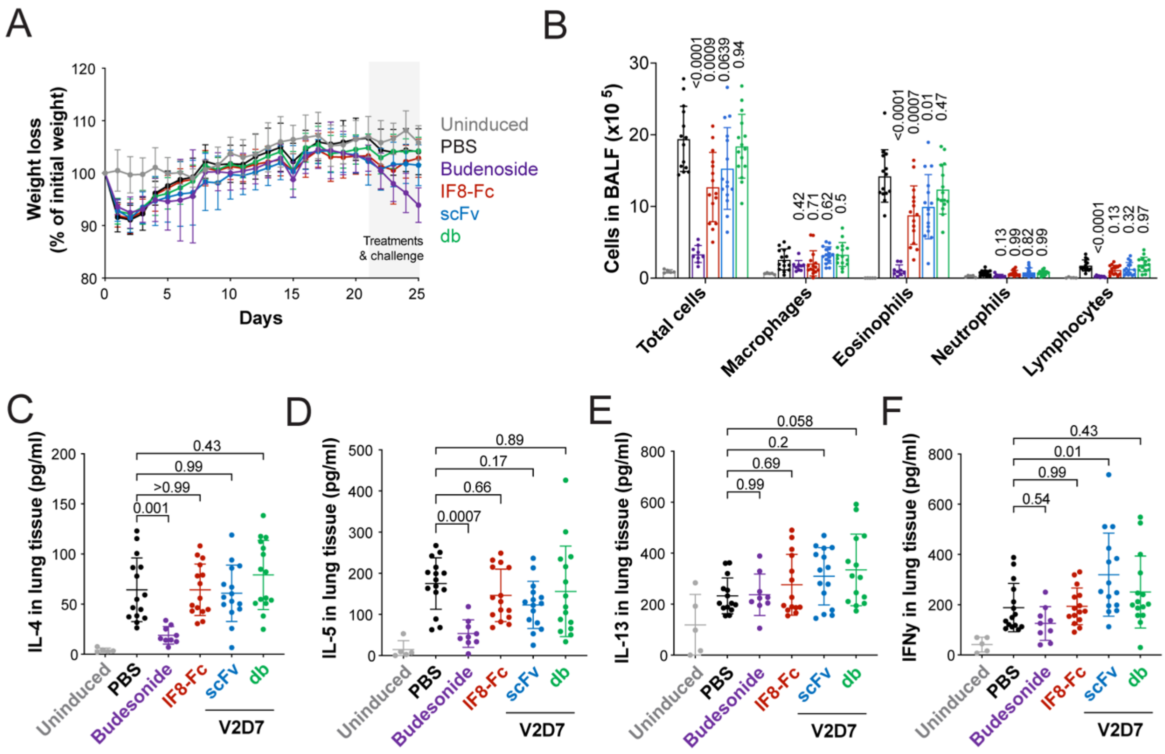

Figure S4: Effects of intranasal treatment with anti-ALCAM antibody fragments or budenoside on immune cell infiltration in a mouse model of asthma.

(A) Measurement of mouse bodyweights over the treatment period. **(B)** Mice sensitized to ovalbumin (OVA) were treated intranasally with vehicle (PBS), budesonide (positive control), IF8-Fc, V2D7scFv, or V2D7db 60 mins prior to OVA challenges (100 µg per dose), following the schedule depicted in Figure 5A. On day 25, mice were sacrificed and the **(B)** number of immune cells in BAL fluid and **(C-F)** concentrations of IL-4, IL-5, IL-13, and IFNγ in lung tissue homogenates were determined. Data are from a single study with 5-15 animals per group. The mean ± SD are shown. Statistics: One-way ANOVA, Dunnett's multiple comparison test (B/C/D/E/F).

1. References:
Raybould, M.I.J.; Marks, C.; Krawczyk, K.; Taddese, B.; Nowak, J.; Lewis, A.P.; Bujotzek, A.; Shi, J.; Deane, C.M. Five computational developability guidelines for therapeutic antibody profiling. *Proc. Natl. Acad. Sci. USA* **2019**, *116*, 4025–4030. https://doi.org/10.1073/pnas.1810576116.
